# The History and Capabilities of Herbal Simples

**Published:** 1892-03-12

**Authors:** W. T. Fernie


### The History and Capabilities of Herbal Simples.

By W. T. Fernie, M.D.

*(Continued from page 4.)*

### XXXVIII.—Feverfew.

Scarcely any plant is more commonly employed by country persons for curative purposes, or more widely found throughout England in hedges, and on waste ground, than the Feverfew. Its name is derived from the Latin word *febrifugus*, putting a fever to flight. This herb is actually the wild chamomile, and grows with numerous small heads of yellow flowers having outermost white rays, but its stem is upright, whereas that of the true chamomile is procumbent. Also it bears the title of *matricaria*, because especially useful for maintaining the health during motherhood. The whole plant has a powerful pungent odour, and is particularly disliked by bees. Its leaves are feathery, and of a delicate green colour, being conspicuous even in midwinter. Chemically the feverfew furnishes a blue volatile oil, which contains a camphoraceous stearoptin, and a liquid hydrocarbon, together with some tannin, and a bitter mucilage. The essential oil is medicinally useful for correcting female irregularities, as well as for obviating cold indigestion. This plant is further called

Mayde-weed, because it is effectual against hysterical distempers, to which virgins are subject. Taken generally, it is found to exercise a positive tonic action on the whole bodily system. In Devonshire the Feverfew is known by the name of Bachelor's Buttons, and at Torquay it is named Flirtwort, being also spoken of sometimes as feathyfew, or featherfall. Dioscorides called it Parthenion, from its efficacy in disorders of women. Our chemists make a medicinal tincture of Featherfew, of which from ten to twenty drops may be taken with a teaspoonful of cold water three times in the day. Gerarde says the plant may be used both in drinks and bound to the wrists, as of singular virtue against the ague. If two teaspoonfuls of the tincture be mixed with half a pint of cold water, and if all parts of the body likely to be exposed to the bites of insects or vermin be freely sponged therewith, no such attacks will be made; also the tincture, if dabbed on in its pure state, will relieve the pain and swelling which ensue from bites inflicted by insects. For internal use the expressed juice may be given medicinally in doses of a tablespoonful at a time, and hot fomentations made from the bruised plant will be found useful for local inflammations. Feverfew is manifestly the progenitor of the *matricaria chamomilla*, from which the highly useful camomile "blows," sold by our druggists for well-known domestic purposes, are now obtained, and its dried flowers may be employed for the same ends. An infusion made from these with boiling water, and allowed to become cold, will allay any distressing sensitiveness to pain in highly nervous subjects, and will afford great relief in ordinary inflammatory or rheumatic faceache. Dr. Guernsey says the feverfew is best calculated to pacify persons of "spiteful, sudden, rude irascibility," of which they are quite conscious, but say they cannot help it; and to tranquillise children who refuse to be soothed save when carried about. King Solomon must have had this amiable plant in his mind when commending a dinner of herbs where love is, before a stalled ox, and hatred therewith.

